# Supplementary material for: The Effect of Non-Invasive Brain Stimulation (NIBS) on Attention and Memory Function in Stroke Rehabilitation Patients: A Systematic Review and Meta-Analysis
Source: Diagnostics (Basel). 2021 Feb 3;11(2):227. doi: 10.3390/diagnostics11020227 (PMC7913379; doi:10.3390/diagnostics11020227)
Supplement: Supplementary file 1 [file diagnostics-11-00227-s001.pdf]

MEDLINE

(("stroke"[MeSH Terms] OR "stroke"[All Fields]) OR ("stroke"[MeSH Terms] OR "stroke"[All Fields] OR ("cerebral"[All Fields] AND "vascular"[All Fields] AND "accident"[All Fields]) OR "cerebral vascular accident"[All Fields]) OR (("ischemia"[MeSH Terms] OR "ischemia"[All Fields] OR "ischemic"[All Fields]) AND ("stroke"[MeSH Terms] OR "stroke"[All Fields])) OR ("intracranial hemorrhages"[MeSH Terms] OR ("intracranial"[All Fields] AND "hemorrhages"[All Fields]) OR "intracranial hemorrhages"[All Fields] OR ("hemorrhagic"[All Fields] AND "stroke"[All Fields]) OR "hemorrhagic stroke"[All Fields])) AND ((non-invasive[All Fields] AND ("Brain Stimul"[Journal] OR ("brain"[All Fields] AND "stimulation"[All Fields]) OR "brain stimulation"[All Fields])) OR ("transcranial magnetic stimulation"[MeSH Terms] OR ("transcranial"[All Fields] AND "magnetic"[All Fields] AND "stimulation"[All Fields]) OR "transcranial magnetic stimulation"[All Fields]) OR (theta-burst[All Fields] AND stimulation[All Fields]) OR (quadripulse[All Fields] AND stimulation[All Fields]) OR ("transcranial direct current stimulation"[MeSH Terms] OR ("transcranial"[All Fields] AND "direct"[All Fields] AND "current"[All Fields] AND "stimulation"[All Fields]) OR "transcranial direct current stimulation"[All Fields] OR ("transcranial"[All Fields] AND "electrical"[All Fields] AND "stimulation"[All Fields]) OR "transcranial electrical stimulation"[All Fields]) OR ("transcranial direct current stimulation"[MeSH Terms] OR ("transcranial"[All Fields] AND "direct"[All Fields] AND "current"[All Fields] AND "stimulation"[All Fields]) OR "transcranial direct current stimulation"[All Fields]) OR ("transcranial direct current stimulation"[MeSH Terms] OR ("transcranial"[All Fields] AND "direct"[All Fields] AND "current"[All Fields] AND "stimulation"[All Fields]) OR "transcranial direct current stimulation"[All Fields] OR ("transcranial"[All Fields] AND "alternating"[All Fields] AND "current"[All Fields] AND "stimulation"[All Fields]) OR "transcranial alternating current stimulation"[All Fields])) AND (("cognition"[MeSH Terms] OR "cognition"[All Fields]) OR ("memory"[MeSH Terms] OR "memory"[All Fields]) OR ("attention"[MeSH Terms] OR "attention"[All Fields]) OR ("executive function"[MeSH Terms] OR

("executive"[All Fields] AND "function"[All Fields]) OR "executive function"[All Fields] OR ("executive"[All Fields] AND "functioning"[All Fields]) OR "executive functioning"[All Fields]))

Scopus

( TITLE-ABS-KEY ( stroke ) OR TITLE-ABS-KEY ( cerebral AND vascular A ND accident ) OR TITLE-ABS-KEY ( ischemic AND stroke ) OR TITLE-ABS-KEY ( hemorrhagic AND stroke ) AND TITLE-ABS-KEY ( non-invasive AN D brain AND stimulation ) OR TITLE-ABS-KEY ( transcranial AND magneti c AND stimulation ) OR TITLE-ABS-KEY ( theta-burst AND stimulation ) O R TITLE-ABS-KEY ( quadripulse AND stimulation ) OR TITLE-ABS-KEY ( tr anscranial AND electrical AND stimulation ) OR TITLE-ABS-KEY ( transcran ial AND direct-current AND stimulation ) OR TITLE-ABS-KEY ( transcrania l AND alternating AND current AND stimulation ) AND TITLE-ABS-KEY ( cognition ) OR TITLE-ABS-KEY ( memory ) OR TITLE-ABS-KEY ( attention ) OR TITLE-ABS-KEY ( executive AND functioning ) )

CINAHL

( Stroke or Cerebral Vascular Accident or Ischemic Stroke or Hemorrhagic Stroke ) AND ( Non-invasive brain stimulation or Transcranial magnetic stimulation or Theta-burst stimulation or Quadripulse stimulation or Transcranial Electrical Stimulation or Transcranial direct-current stimulation or Transcranial Alternating current stimulation ) AND ( Cognition or Memory or Attention or Executive functioning )

Embase

((Stroke or Cerebral Vascular Accident or Ischemic Stroke or Hemorrhagic Stroke) and (Non-invasive brain stimulation or Transcranial magnetic Stimulation or Theta-burst stimulation or Quadripluse stimulation or Transcranial Electrical Stimulation or Transcranial direct-current stimulation or Transcranial Alternating current stimulation) and (Cognition or Memory or Attention or Executive functioning)).mp. [mp=title, abstract, heading word,

drug trade name, original title, device manufacturer, drug manufacturer, device trade name, keyword, floating subheading word, candidate term word]

PsycINFO
noft((Stroke OR Cerebral Vascular Accident OR Ischemic Stroke
OR Hemorrhagic Stroke) ) AND noft((Non-invasive brain stimulation OR Transcranial magnetic
Stimulation OR Theta-burst stimulation OR Quadripluse stimulation OR Transcranial
Electrical Stimulation OR Transcranial direct-current stimulation OR Transcranial
Alternating current stimulation)) AND noft((Cognition OR Memory OR Attention OR Executive functioning))

CENTRAL
(Stroke OR Cerebral Vascular Accident OR Ischemic Stroke OR Hemorrhagic Stroke) AND (Non-invasive brain stimulation OR Transcranial magnetic Stimulation OR Theta-burst stimulation OR Quadripulse stimulation OR Transcranial Electrical Stimulation OR Transcranial direct-current stimulation OR Transcranial Alternating current stimulation) AND (Cognition OR Memory OR Attention OR Executive functioning) in All Text - (Word variations have been searched)
